# Alcohol use and abuse in training conscripts of the Hellenic navy

**DOI:** 10.1186/1744-859X-5-21

**Published:** 2006-11-29

**Authors:** Georgios Moussas, Leonidas Tzemos, Vassilis Pavlopoulos, Konstantinos Papadimitriou, Vassilis Menoutis, Lefteris Lykouras

**Affiliations:** 1 Secont Psychiatric Department Medical School University of Athens "Attikon" General Hospital, Athens, Greece; 2 Office of Preventive Mental Health, Hellenic Navy, Skaramangas, Greece; 3 Department of Psychology, University of Athens, Athens, Greece; 4 Athens Naval Hospital , Hellenic Navy , Athens, Greece

## Abstract

**Objectives:**

Alcohol abuse and addiction are big current problems of the developed world having multivariate causality and multiple effects. Alcohol abuse in young people is a matter of central importance due to its wide range long lasting effects, especially so in Greece where the problem has only recently started growing. The Hellenic Navy is interested in the complications of alcohol abuse in training conscripts. Because young conscripts will be placed in demanding positions, but also because in Greece the military service is obligatory and represents an important period for the socialization of young men.

**Methods:**

In the present study, levels of alcohol use and abuse were measured in a sample of 650 male training conscripts of the Hellenic Navy. The tools used are: (a) two questionnaires measuring frequency and quantity of alcohol consumption and psychosocial variables, (b) the CAGE test, which is a questionnaire measuring hidden alcoholism.

**Results:**

38,1% conscripts were characterized problematic drinkers according the adolescents criteria. Additional psychological complications were related to alcohol use. Using the stricter criterion for adults (plus psychological complications) 8.9% were found to be problematic drinkers. The use of CAGE questionnaire which is measuring hidden alcoholism, identified 16% of the total sample as hidden alcoholics.

**Discussion:**

The findings regarding unregular levels of alcohol use and abuse are presented as well as their relation to psychosocial complications and to demographic characteristics. The results are discussed in the light of Creek and international bibliography.

## Background

Alcoholism is considered to be a very important problem that has taken huge dimensions since World War II [1]. There is a growing recognition of the significant contribution of alcohol to the global burden of illness, disability and death [2]. Europe holds the highest position in alcohol consumption rates and health problems rates connected with alcohol [3]. Alcohol (ethanol) abuse and dependence are the most common substance use disorders among adolescents [4]. Until recently in Greece, the problem of alcohol use and abuse was considered non existent by the society and health professionals, with the exception of specialists dealing with substance use problems.

Recent evidence, however, has changed the picture. Such evidence comes from studies that have shown an increase in per capita consumption of pure ethanol as well as a change in the traditional manner of alcohol consumption the last two decades compared with previous consumption [5-8]. Further evidence comes from identifying alcohol use and abuse, in general hospitals inpatients [9-11] as well as from studies in the general population [12,13]. Alcohol consumption and harm indicators like hospital discharges, injury and poisoning per 100.000 populations, classify Greece 17^th ^in a total of 52 countries [14]. Studies of the Athens University Mental Health Research Institute showed a rise in the rates of students presenting a very frequent consumption of alcoholic drinks. The mean age of onset of alcohol consumption in Greece appears to be similar with that of 35 other countries [6,7].

Alcohol abuse among adolescents and its complications are considered to be major public health issues of the developed world [15-18], since alcohol abuse in adolescents and young adults is known to be related to high risk for life loss [19-22]. Alcohol abuse is especially known to play a major role in traffic accidents (fatalities and injury accidents). Greece has one of the highest rates of fatal traffic accidents among the countries of the European Union [23]. According to a recent study 45% of lethal traffic accidents in Greece are related to alcohol concentration of 0.5 g/l [24].

Training conscripts of the Hellenic Navy can be considered to be a good sample of healthy young males as their ages normally falls between 18 and 24 years. However, about 10–20% of the conscripts are usually older than 25 years [25,26]. Thus, the diversity in the age range of conscript personnel in the Armed Forces, made the use of both questionnaires compulsory on older adolescents and young adults into separation the two age subgroups was not feasible for technical reasons (as for the age mixed military units), on the other hand any research studying alcohol consumption among such a sample should be very carefully designed. The tools used to measure alcohol consumption of adolescents/young adults are different from those used in older age groups [13]. It is very important for the Hellenic Navy to have a profile of alcohol use and abuse of the training conscripts, firstly because the Navy is responsible for the conscripts' health while they are in service, and secondly because the conscripts are placed in responsible and demanding positions. It is also known that the period of obligatory military service is a high stress one and alcohol abuse and stress are related to violence, suicidality and self harm acts [19,27].

The aim of the present study is a twofold one, i.e. to measure problematic drinking among training conscripts of the Hellenic Navy both by adult and by adolescent standards, and to measure levels of hidden alcoholism in the same sample. Problematic drinking is a bi-axial measure involving frequency and quantity of alcohol use, along with psychosocial complications related to alcohol use. Problematic alcohol use is considered to be a predictor for future alcohol abuse and/or addiction problems. The consequence of "problematic use" is gradual habituation on pathological use that is not appears, because of social culture and social culture and youth habits [13]. Hidden alcoholism is because of an indirect measure of existing alcohol abuse and/or addiction, which is employed in order to avoid the large number of false negative response produced by traditional tools measuring alcoholism [28].

## Method

### Sample

Six hundred and sixty (660) training conscripts of the Hellenic Navy participated in the study. The subjects' age ranges from 18 to 37 years (Mn = 22.0, St.dev = 2.8). Almost 20% (n = 118) were older than 25 years of age. They came from different parts of Greece (63.8% from Athens, Thessaloniki or other big cities; 25.6% from little towns or villages; and 10.6% from islands). Their education varies from basic – 6 years – (8.0%), and secondary – 12 years – (17.3%), to technical/professional – 9 years – (39.1%), and University – 16 or more years – (35.5%). Only 18 (2.7%) conscripts were married, while the parents of 50 (7.6%) were separated or divorced. It must be noted that all subjects were qualified as being capable of serving the Armed Forces (subjects suffering from serious physical or psychiatric diseases had already received a deferment or a discharged).

### Measures and procedure

The following tools were used, (a) a questionnaire measuring frequency and quantity of alcohol consumption in adults [see Additional file 1], [13] (b) a questionnaire measuring frequency and quantity of alcohol consumption in adolescents [see Additional file 1], [13] (c) a questionnaire assessing psychosocial complications related to alcohol use [see additional file 1], [13]. The two questionnaires measuring frequency and quantity of alcohol consumption in adolescents and in adults were used in the total of our sample as already mentioned, for the following reasons: In order to cover the wide range of diverse age of controls (age 18 to 35 years old) and to cope with our technical inability to separate our subjects as for the age – mixed military units. The questionnaires comprising frequency/quantity questions are suitable for the detection of alcohol related problems as suggested by the WHO 2004 [8], and also by studies referring to the alcohol abuse problems [13,29,30]. The reliability of the questionnaires of frequency and quantity of alcohol consumption has been tested by the estimation of their interreliability coefficient kappa of agreement. The questionnaires were applied to a number of 50 alcohol dependent subjects by the two undependent rates. Their agreement was satisfactory and kappa was 0.92. (d) The CAGE test [see additional file 1], that traces hidden alcoholism [28,31] and which is suitable for the detection of non hazardous and harmful alcohol consumption [32] which is used in everyday practice and research [33,34]. The above questionnaire has been translated and standardized in Greek population [35]. The score assigned to each participant for each questionnaire represents the number of positive responses given by the participant in the respective test. In addition, the subjects provided information concerning demographic characteristics.

The collection of data took place at the training campus of the Hellenic Navy on the island of Poros in November 1998. Groups of 20–30 people completed the questionnaires in the presence of a psychiatrist and two psychologists trained in questionnaire administration. All conscripts participated in the study on a voluntary basis, after they had been informed about the general purpose of the study.

## Results

### Problematic drinking

A participant was considered to be a problematic drinker according to *adolescent *standards when he obtained a score of one or higher in the frequency/quantity questionnaire for adolescents *plus *a score of one or higher in the questionnaire measuring psychosocial complications related to alcohol use [13]. Using this criterion, 205 (31.8%) conscripts are characterized as problematic drinkers (Table 1).

**Table 1:** Frequency of problematic drinkers by means of adolescent criteria (adolescents' scale score ≥ 1 plus 1 psychosocial variable)

Frequency/quantity of alcohol use	*f*	Valid %
Normal use	440	68.2
Problematic drinking	205	31.8
Total	645	100.0

Missing cases: 15 (2.3%)

A participant was considered to be a problematic drinker according to *adult *standards when he obtained a score of two or higher in the frequency/quantity questionnaire for adults *plus *a score of two or higher in the questionnaire measuring psychosocial complications related to alcohol use [13]. Using this stricter criterion, 57 (8.9%) subjects were found to be problematic drinkers (Table 2).

**Table 2:** Frequency of problematic drinkers by means of adult criteria (adolescents' scale score ≥ 2 plus 2 psychosocial variables)

Frequency/quantity of alcohol use	*f*	Valid %
Normal use	584	91.1
Problematic drinking	57	8.1
Total	641	100.0

Missing cases: 19 (2.9%)

As expected, scores in the adolescent and in the adult questionnaires on frequency/quantity of alcohol consumption were highly intercorrelated (Pearson's r = 0.56, p < .01). However, correlation of score in the psychosocial complications measure with the scores in each of the two alcohol consumption measures produced considerably lower, though still significant, coefficients: Pearson's r = 0.22 (p < .01) for the adults' questionnaire; and Pearson's r = 0.28 (p < .01) for the adolescents' questionnaire.

### Hidden alcoholism

A participant was considered to be a hidden alcoholic when he scored two or higher in the CAGE test [28,31,35]. According to this criterion, 101 (16.4%) subjects were found to be positive (Table 3).

**Table 3:** Scores in the CAGE test measuring hidden alcoholism

**Category**	**Score**	***f***	**Valid %**	**Cum %**
Normal	0	394	64.1	64.1
	1	120	19.5	83.6

Hidden	2	66	10.7	94.3
alcoholism	3	29	4.7	99.0
	4	6	1.0	100.0

	Total	615	100.0	

Missin cases: 45 (6.8%)

A positive correlation coefficient was found between the score in the CAGE test and the score in the psychosocial complications questionnaire (Pearson's r = .35, p < .01). In accordance to the previous finding, when hidden alcoholics were compared to non hidden alcoholics in terms of their scores on the psychosocial complications questionnaire, the former gave significantly more positive responses than the latter in seven (out of the eight) psychosocial complications related to alcohol (Table 4).

**Table 4:** Psychosocial variables related to alcohol use by hidden alcoholism

**Psychosocial variables**	**Hidden alcoholism**					
	**No**		**Yes**		**Total**	
	***f***	**Valid %**	***f***	**Valid %**	***f***	**Valid %**
Drink when in bad mood	121	23.6**	50	49.5**	171	27.9
Alcohol use depresses me	48	9.4*	17	16.8*	65	10.6
Accident after alcohol use	39	7.6**	21	20.8**	60	9.8
Strong need for alcohol	36	7.0**	15	14.9**	51	8.3
Work problems	31	6.0*	12	11.9*	43	7.0
Family problems	12	2.3**	16	15.8**	28	4.6
Study/school problems	6	1.2**	11	10.9**	17	2.8
Drink alone or first thing	10	1.9	5	5.0	15	2.4

*p < .05; **p < .01

On the other hand, correlation of scores in the CAGE test with the scores of questionnaires measuring problematic drinking in adults and adolescents produced rather low coefficients: Pearson's r = .27, p < .01, and r = .30, p < .01, respectively.

## Discussion

The main finding of this study is that almost one third (31.8%) of the training conscripts were found to be problematic, alcohol drinkers according to adolescent standards which probably lends support to the current view that there is a trend of the Greek youth towards alcohol consumption.

This finding may be of importance as has been demonstrated that once the problematic alcohol use can be detected and modified at an earlier stage, then alcohol related problems can be prevented [36,37].

Even when the adult criterion was used, which is generally stricter and applies to samples of older age [13], about one in ten subjects (8.9%) still scored positively in problematic alcohol drinking. The above high percentages are in accordance with the results of other studies among young people in Greece [38] and the increasing frequency of binge (which means that there is a consume of great quantities of alcohol in a few hours) drinking among Greek adolescents [6,7]. These findings may indicate that a underlying problem of alcohol abuse exists among training conscripts of the Hellenic Navy, since the questionnaire used include items giving information about the amount and frequency of alcohol that is in fact binge drinking. Since the questionnaires used provide indirect information about binge drinking habit in conscript behaviour.

The high level of problematic drinking cannot be attributed to conditions within the Navy as the testing of the conscripts was carried out training period i.e. second and third week. During that time the conscripts stay continuously in the enlistment camp and have no access to alcohol.

However, the oncoming enlistment in the Armed Forces can be perceived by the conscript as a stressful life event and stressful events are known to be related to increased alcohol consumption [39-41] as was shown with the high scores in the frequency/quantity questionnaire. Since the high scores in the psychosocial complication questionnaires indicate longterm abuse and not a circumstancial increase in alcohol consumption. Furthermore one should have in mind, the possible coexistence of other psychiatric disorders, those sorts of disorders were not detected in the present study [36,42,43].

Since training conscripts are representative samples of the healthy young males in Greece because military service in Greece is obligatory for the entire male population. Furthermore the distribution to different military corps is random, the conscript come urban, semi-urban and rural areas and they are of all educational levels and socioeconomic conditions. In this regard training conscripts could be considered as a representative sample of the health males in Greece and the present results can be generalized to the population. Thus, our findings support the recent view that problematic alcohol use in Greece is high [5].

An important finding is that 16% of the participants were shown to be positive in hidden alcoholism according the CACE questionnaire, and it is very closed to the percentage of alcohol consumption among Greek physical education students, indicated a percentage 17,31%, with the same questionnaire [44]. Even if we consider that about 10% of the sample response false positive answers in the CAGE test [28,31,35] the remaining 14.3% percentage of hidden alcoholism is significant.

The validity of CAGE test has been established in numerous studies detecting alcohol abuse in the General Hospital [33,34] and in the classification detection and diagnosis is chronic alcoholic disorder [45].

The percentage of hidden alcoholism in Greek Navy conscripts is within the prevalence range (12.5–30%0 of alcohol related problems found in the general hospital inpatients [10,9]. The prevalence of alcohol related-problems in general hospitals range from 12.5% to 30% [46]. The present study findings need to be confirmed by others studies in the community probably with the use of different questionnaires and biological parameters as well.

On the whole, the results support the view that alcohol use and abuse has risen to a major health problem in Greece [12,13]. Recent studies have proved that the increase in alcohol abuse may be of critical importance as it is related to psychosocial stresses, and could associate with suicidal behaviour or parasuicide behaviour [47,48]. They also show that the Hellenic Navy is not immune to health problems of the community; and thus stress the need for the existence of structures in the Hellenic Navy that can investigate the extent and nature of such problems and produce educated proposals for their solution and prevention.

## Conclusion

The results of this study for the an regular and problematic relation with alcohol consumption of the Hellenic Navy conscripts must be furthermore studies in order to confirm the findings in this critical groups of ages, because this group go through a traditional phase in their lives, and the results of this kind must be re-examined.

## Supplementary Material

Additional File 1 Questionnaires. The questionnaires detect hidden alcoholism related to psychosocial parametres. Click here for file
