# Supplementary material for: Alcohol use and abuse in training conscripts of the Hellenic navy
Source: Ann Gen Psychiatry. 2006 Nov 29;5:21. doi: 10.1186/1744-859X-5-21 (PMC1698475; doi:10.1186/1744-859X-5-21)
Supplement: Additional File 1 — Questionnaires. The questionnaires detect hidden alcoholism related to psychosocial parametres. [file 1744-859X-5-21-S1.doc]

**CAGE Questionnaire**

- Have you ever left you should **C**ut down on your drinking?
- Have people **A**nnoyed you bay criticizing your drinking?
- Have you ever left bad or **G**uilty about your drinking?
- Have you ever had a drink first thing in the morning to steady your nerves or to get rid of a hangover (**E**ye opener)?

To find criteria for identifying problematic drinking in adolescents, a typology of consumption was developed including: (a) frequency and quantity of alcohol use and B) a list of psychosocial problems related to excessive drinking.

**Title: Frequency/quantity Questionnaire for adolescents**

**Description: The questionnaire detects alcohol abuse in adolescents**

A) Frequency and Quantity of Questionnaire Adolescents alcohol use:

|  |  | YES | NO |
| --- | --- | --- | --- |
| 1 | Every day alcohol use (>1 times), the last 30 days |  |  |
| 2 | Everyday alcohol use during the last week |  |  |
| 3 | Episode of drunkenness (got drug almost every time they drunk or they got drunk >3 times in their lifetime) |  |  |

**Title: Frequency/ quantity questionnaire for adults**

**Description: The questionnaire detects alcohol abuse in adults**

B) Adults questionnaire frequency and quantity alcohol use:

|  |  | YES | NO |
| --- | --- | --- | --- |
| 1 | Have left dizzy most of the times they drank |  |  |
| 2 | Got drunk or lost of control 6-9 times |  |  |
| 3 | Have drunk 9 or more drinks in one day for 2 or more days |  |  |
| 4 | 6-8 drinks for 2 or more days or 3-5 drinks for 3 or more days |  |  |

**Title: List of psychosocial problems**

**Description: The list investigates psychosocial problems related to alcohol abuse**

**C) A list of psychosocial problems relat**ed to excessive drinking:

| 🞏 | Compulsive drinking |
| --- | --- |
| 🞏 | Reported drinking to overcome other problems |
| 🞏 | Drink when in had mood |
| 🞏 | Drink alone or first thing in the morning |
| 🞏 | Alcohol use has caused problems with family or police |
| 🞏 | Alcohol use has caused problems in work or school friends |
| 🞏 | A traffic accident |
| 🞏 | Psychological problems or impairment in physical health |
